# VST-DAVis: an R Shiny application and web-browser for spatial transcriptomics data analysis and visualization

**DOI:** 10.1093/bioadv/vbag007

**Published:** 2026-01-09

**Authors:** Sankarasubramanian Jagadesan, Chittibabu Guda

**Affiliations:** Department of Genetics, Cell Biology and Anatomy, 985805 Nebraska Medical Center, University of Nebraska Medical Center, Omaha, NE 68198-5805, United States; Department of Genetics, Cell Biology and Anatomy, 985805 Nebraska Medical Center, University of Nebraska Medical Center, Omaha, NE 68198-5805, United States; Center for Biomedical Informatics Research and Innovation, 985805 Nebraska Medical Center, University of Nebraska Medical Center, Omaha, NE 68198-5805, United States

## Abstract

**Summary:**

Visium HD Spatial Transcriptomics Data Analysis and Visualization (VST-DAVis) is an interactive, R Shiny application and web browser designed for intuitive analysis of spatial transcriptomics data generated using the 10x Genomics Visium HD platform. This user-friendly tool empowers researchers, particularly those without programming expertise, to perform end-to-end spatial transcriptomics analysis through a streamlined graphical interface. The platform is capable of handling both single and multiple samples, enabling comparative analyses across diverse biological conditions or replicates. It accepts various input formats including both H5 and matrix-based files from Space Ranger and outputs high-quality graphics from various visualization tools. VST-DAVis integrates several widely used R packages, such as Seurat, Monocle3, CellChat, and hdWGCNA, to offer a robust and flexible analytical environment that supports a wide range of analytical tasks, including quality control, clustering, marker gene identification, subclustering, trajectory inference, pathway enrichment analysis, cell–cell communication modeling, co-expression analysis, and transcription factor network reconstruction. By combining its analytical depth with user-friendliness, VST-DAVis makes advanced analyses accessible to various research communities that utilize spatial transcriptomics data.

**Availability and implementation:**

VST-DAVis is freely available at https://www.gudalab-rtools.net/VST-DAVis. It is implemented in R 4.5.2 and Bioconductor ≥ 3.22 using the Shiny framework and supports input from Space Ranger outputs. The source code and documentation are hosted on GitHub: https://github.com/GudaLab/VST-DAVis.

## 1 Introduction

Spatial transcriptomics has transformed our ability to study gene expression within the intact tissue architecture by allowing for a deeper understanding of complex tissues in healthy and disease states. By retaining spatial relationships, the effects of gene expression can be studied at the cellular microenvironments, which might not be captured by single-cell RNA sequencing. The recently introduced 10x Genomics Visium HD provides single-cell level resolution, enabling unprecedented exploration of cellular heterogeneity and spatial organization. However, comprehensive analysis of these high-resolution datasets remains challenging due to the dearth of complex computational workflows (Ståhl *et al.* 2016). While powerful tools like Seurat (Hao *et al.* 2024), Giotto (Dries *et al.* 2021), STUtility (Bergenstråhle *et al.* 2020), and Squidpy (Palla *et al.* 2022) offer comprehensive solutions for spatial clustering, single-cell integration, and spatial gene expression analysis, their reliance on command-line interfaces and coding expertise creates barriers for experimental biologists (Moses and Pachter 2022). Existing web-based platforms such as webSCST (Zhang *et al.* 2022), SpatialView (Mohanty *et al.* 2024), SpatialDE (Svensson *et al.* 2018) have partially addressing this issue but are limited in their ability to simultaneously process multiple samples and perform advanced clustering analyses. spatialLIBD (Maynard *et al.* 2021) is designed for interactive exploration of preprocessed brain datasets. However, it only allows users to analyze the datasets provided on the platform and does not support uploading or analyzing custom datasets through a web interface. To overcome these limitations, we developed Visium Spatial Transcriptomics Data Analysis and Visualization (VST-DAVis) standalone software and an intuitive web application that combine analytical robustness with user-friendly visualization, enabling researchers at all computational skill levels to perform end-to-end spatial transcriptomics analyses and generate publication-quality results.

## 2 Features and functionality

**Figure 1.**
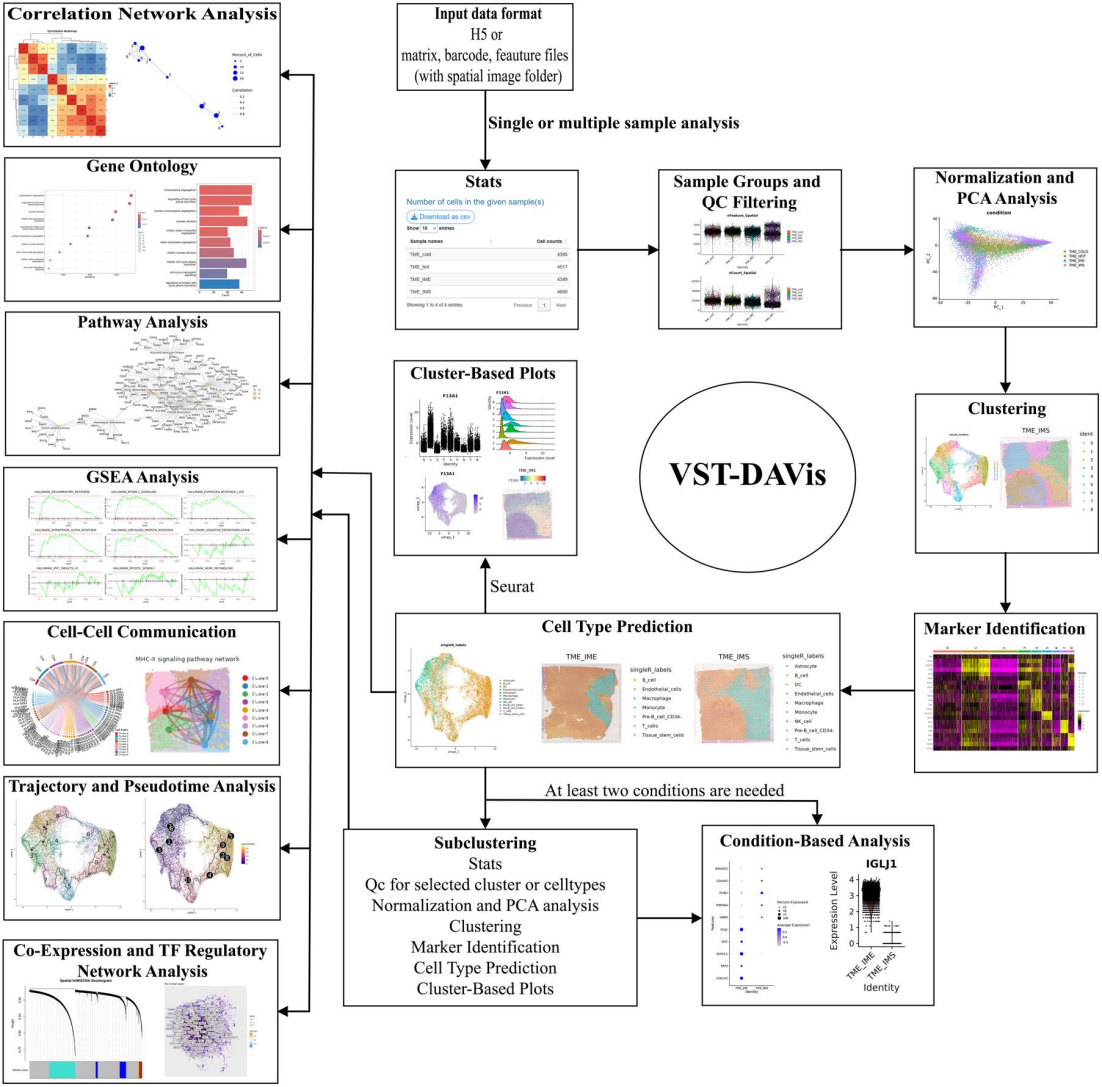
Overview of VST-DAVis workflow. Figure depicts the nine integrated analytical modules showing the sequential steps, inputs, outputs, and interconnectivity among the modules.

VST-DAVis supports a wide range of functionalities: It accepts both H5 (filtered_feature_bc_matrix.h5) and Matrix formats (matrix.mtx.gz, features.tsv.gz, barcodes.tsv.gz) with spatial image folder from 10x Space Ranger output. Users who download sample files from NCBI or other public repositories need to rename and organize their files to match the Space Ranger output format, which is required by our application. To support this, we have provided clear and detailed upload instructions on the upload page, including a reference image that illustrates the correct directory structure. Users can also download and extract example datasets directly from our GitHub repository or the application’s upload page. These examples will help users properly format their data and ensure full compatibility with our tool. Different analytical features are embedded into nine functionally related modules that include: (i) Single or Multiple Sample Analysis, (ii) Subclustering, (iii) Correlation Network Analysis, (iv) Genome Ontology Analysis, (v) Pathway Analysis, (vi) Gene Set Enrichment Analysis, (vii) Cell–Cell Communication Analysis, (viii) Trajectory and Pseudotime Analysis, and (ix) Co-Expression and TF analysis. Following initial data processing in Module 1 using a single or multiple samples, users can seamlessely execute subsequent modules in any order. Figure 1 graphically illustrates the implemented modules in the VST-DAVis platform. The full list of packages used to develop the application is provided in Table 1, available as supplementary data at *Bioinformatics Advances* online. VST-DAVis provides a comprehensive spatial transcriptomics analysis pipeline, starting with preprocessing and quality control. The platform performs quality assessment using Seurat, offering both LogNormalize and SCTransform (Hafemeister and Satija 2019) normalization methods. For downstream analysis, it incorporates standard dimensionality reduction techniques (PCA, UMAP, t-SNE) coupled with Seurat-based clustering algorithms, including Louvain, SLM, Leiden. Cluster-specific marker gene identification is enabled through customizable differential expression parameters (cluster-specific markers, markers in one or a few clusters, conserved markers), followed by flexible cell type annotation using tools like ScType (Ianevski *et al.* 2022), SingleR (Aran *et al.* 2019), GPTCelltype (Hou and Ji 2024), or manual labeling. The platform offers versatile subclustering functionality through multiple intuitive approaches. Users can also perform targeted subclustering analysis by (i) selecting specific clusters from the initial Seurat-based clustering results, (ii) isolating cell populations based on predicted cell type annotations, or (iii) dynamically subsetting data using marker gene expression patterns. The platform provides flexibility for focused analyses by enabling both positive selection (extracting cells expressing genes of interest) and negative selection (excluding specified cell populations while retaining all others) for downstream characterization. This multi-modal subclustering capability allows users to investigate rare cell populations or focus on specific cellular compartments within a complex tissue. Advanced functional modules offer trajectory inference and pseudotime analysis via Monocle3 (Cao *et al.* 2019), pathway and Gene Ontology term enrichment analysis using clusterProfiler (Yu *et al.* 2012), ReactomePA (Yu and He 2016) and fgsea (Korotkevich *et al.* 2016), and cell–cell communication prediction with CellChat (Jin *et al.* 2025). The platform also implements high-dimensional weighted gene co-expression network analysis (hdWGCNA) (Morabito *et al.* 2023) for network biology applications to identify co-expression modules and reconstruct transcription factor regulatory networks. All adjustable parameters used in the VST-DAVis web application are detailed in Table 2, available as supplementary data at *Bioinformatics Advances* online. We provide Table 3, available as supplementary data at *Bioinformatics Advances* online, which compiles the command-line tools and their tutorials for all packages combined into VST-DAVis, supporting QC-to-advanced analyses without coding. Importantly, all these advanced analyses can be performed using the complete dataset or any user-defined subset, including Seurat clusters, predicted cell types, or custom subpopulations selected through marker gene expression. Module 1 provides the basic and essential functionality for typical spatial data analysis projects, while modules 2 to 9 offer additional and advanced functionality that should be invoked only if appropriate to the study design of the project. Advanced modules are opt-in only, not required.

## 3 Visualization

VST-DAVis is built entirely in R (version 4.5.2 or later), by leveraging Shiny (version 1.11.1 or later) for interactivity, and DT, ggplot2, plotly, Seurat and ComplexHeatmap for visual outputs. It is optimized for browser usage and runs locally or on remote servers without requiring command-line operations, similar to our prior work on developing R Shiny applications like MetaDAVis (Jagadesan and Guda 2025a) and ScRDAVis (Jagadesan and Guda 2025b). Outputs can be exported in high-resolution graphic formats including JPG, TIFF, PDF, SVG, BMP, EPS, and PS. Summary tables are displayed using the DataTables (DT) package, allowing up to 100 rows to be viewed interactively, while the complete tables can be downloaded as .csv files.

## 4 Use cases

We validated VST-DAVis using two publicly available high-resolution spatial transcriptomics datasets: GSE230207 (Xia *et al.* 2023), which profiles the tumor microenvironment across immune subtypes in primary central nervous system lymphoma (in H5 format with spatial images), and GSE244014 (Kaplan *et al.* 2024), which provides matrix-format data (matrix.mtx.gz, features.tsv.gz, barcodes.tsv.gz) with spatial images of embryonic mouse diaphragm muscle. The analysis pipeline begins with quality control, where key metrics are computed and visualized across spatial coordinates to identify potential technical artifacts or low-quality spots. Following QC, the data is normalized using SCTransform, enabling downstream dimensionality reduction (PCA) and visualization (UMAP). Users can perform clustering at customizable resolutions to reveal spatially organized cell populations enriched with biologically relevant gene signatures. For each cluster, VST-DAVis identifies marker genes and supports cell type annotation through reference-based methods such as SingleR prediction. The analytical workflow extends to advanced downstream analyses, including: (i) trajectory inference and pseudotime analysis using Monocle3 to reconstruct cellular dynamics; (ii) spatial enrichment of Gene Ontology terms and pathways to uncover region-specific biological processes; (iii) cell–cell communication analysis using CellChat to map potential ligand-receptor interactions between neighboring clusters; and (iv) co-expression network construction via hdWGCNA to identify hub genes, transcription factors, and functional modules associated with distinct cellular states. Throughout the workflow, VST-DAVis generates publication-ready visualizations, which can be exported in various high-resolution formats. The tool was tested on both Linux (RedHat and Ubuntu) and Windows (10 and 11) operating systems. Users can explore the tool with default parameters by selecting the example dataset GSE230207 from the dropdown menu on the upload page. A detailed user manual is also available in the “Manual” tab of the application.

## 5 Conclusion

VST-DAVis is a software tool that addresses a critical need for an open-source, user-friendly, high-resolution spatial transcriptomics data analysis platform, which can be used by researchers without any programming expertise. Its modular architecture, compatibility with multiple data formats, and integration with advanced third-party functional analysis tools provide users with the flexibility to tailor workflows to their specific research questions. The platform’s ability to support multi-sample comparisons, dynamic subclustering, and network-level insights makes it particularly well-suited to carry out deep analytics on spatial transcriptomics datasets. By enabling reproducible, publication-ready graphical outputs without the need for coding, VST-DAVis enhances the pace and quality of spatial transcriptomics research across a broad spectrum of biomedical domains. In the future, we intend to add new functionality to VST-DAVis to support additional input data formats and carry out more downstream analysis tasks.

## Supplementary Material

vbag007_Supplementary_Data

## Data Availability

VST-DAVis is freely available at https://www.gudalab-rtools.net/VST-DAVis. The source code and documentation are hosted on GitHub: https://github.com/GudaLab/VST-DAVis.
