# Supplementary material for: VST-DAVis: an R Shiny application and web-browser for spatial transcriptomics data analysis and visualization
Source: Bioinform Adv. 2026 Jan 9;6(1):vbag007. doi: 10.1093/bioadv/vbag007 (PMC12866912; doi:10.1093/bioadv/vbag007)
Supplement: vbag007_Supplementary_Data [file vbag007_supplementary_data.zip › Table_S2.docx]

**Table S2**. Description of various parameters used in VST-DAVis

This supplementary table summarizes all adjustable parameters available in the VST-DAVis web application. Each parameter is linked to specific analytical modules, including marker identification, cell type prediction, plot customization, correlation and enrichment analysis, trajectory inference, co-expression modules, and transcription factor regulatory networks. Default values and brief descriptions of their function are included to support reproducibility and interpretation of the analysis workflows.

| **Parameter Name** | **Default Value** | **Description** |
| --- | --- | --- |
| **File upload and Stats** | | |
| Upload H5 File (Cell Ranger Output) | H5 | Rename it to SAMPLE_1.zip for proper identification. Contains filtered_feature_bc_matrix.h5 and spatial folder from spaceranger output |
| Upload Matrix files (mtx, features, barcodes) | Matrix | Rename as SAMPLE_1.zip. Contains filtered_feature_bc_matrix folder with matrix.mtx.gz, features.tsv.gz, and barcodes.tsv.gz, and spatial folder from spaceranger output. |
| **Sample Groups and QC Filtering** | | |
| Number of groups | 1 to 6 | Select up to 6 groups. |
| Group 1 Name | Group1 | Type the group name. |
| Group 2 Name | Group2 | Type the group name. |
| Group 3 Name | Group3 | Type the group name. |
| Group 4 Name | Group4 | Type the group name. |
| Group 5 Name | Group5 | Type the group name. |
| Group 6 Name | Group6 | Type the group name. |
| Min gene count per cell | 0 | Filters out cells with fewer than this number of genes expressed.[Recommended: 200 to 500] |
| Max gene count per cell | 10000 | Filters out cells with more than this number of genes expressed. [Recommended: 5000 to 10000] |
| Max mitochondrial % | 5 | Removes cells with excessive mitochondrial gene expression, often indicating low-quality or dying cells. [Recommended: <10%]. |
| **Normalization and PCA Analysis** | | |
| Scale factor | 10000 | Scale factor used in LogNormalize method for total expression normalization. Min: 1, Max: 1e6 |
| Variable gene method | vst | Method for selecting variable features: vst (default), mean.var.plot, or dispersion. |
| Number of variable genes | 2000 | Number of top variable genes to retain for downstream analysis. Min: 100, Max: 10000 |
| PCA dimensions | 30 | Number of principal components computed for dimensionality reduction. Min: 2, Max: 100 |
| Integration method | CCAIntegration | Data integration method. |
| CCAIntegration | Reduction = cca; Distance = Euclidean | Canonical correlation analysis for dataset integration. |
| RPCAIntegration | Reduction = rpca; Distance = Euclidean | Faster, scalable variant of CCA. |
| **Clustering** | | |
| Clustering resolution | 0.5 | Resolution used for cluster granularity. Higher = more clusters. Min: 0.1, Max: 1 |
| Clustering algorithm | Louvain | Graph-based clustering algorithm: Louvain (1), SLM (3), or Leiden (4). |
| UMAP k-nearest-neighbours | 20 | Number of nearest neighbors considered for UMAP. Min: 2, Max: 50 |
| UMAP dims | 30 | Number of PCs used for UMAP dimensionality reduction. Min: 2, Max: 100 |
| UMAP min.dist | 0.3 | Controls how tightly UMAP clusters points. Smaller = more tightly packed. Min: 0.001, Max: 0.5 |
| tSNE dims | 30 | Number of PCs used for t-SNE dimensionality reduction. Min: 2, Max: 100 |
| **Marker Identification** | | |
| FindAllMarkers | Select all cluster | Identifies marker genes for each cluster compared to all other cells. |
| FindMarkers | Select one cluster against another cluster | Finds differentially expressed genes between two specific groups of cells. |
| FindConservedMarkers | Select one cluster to check for conserved in all clusters | Identifies markers that are conserved across multiple groups (e.g., conditions or batches). |
| min.pct | 0.25 | Minimum fraction of cells expressing the gene for it to be tested. Min: 0.01, Max: 1.0 |
| logfc.threshold | 0.25 | Minimum log fold change required to consider gene differentially expressed. Min: 0.01, Max: ∞ |
| Statistical test | wilcox | Statistical test used for differentially expressed gene or marker identification. |
| Return only positive markers | Yes | Whether to return only genes upregulated in the target group. |
| **Cell Type Prediction** | | |
| Cell type method | SingleR | Methods for cell type prediction. ScType, SingleR, GPTCelltype, Use Own Labels |
| Reference tissue for SingleR | hpca | Reference data sources for SingleR annotation. hpca, blueprint_encode, mouse_rnaseq, immgen, dice, novershtern_hematopoietic, monaco_immune |
| DE method for SingleR | classic | SingleR Differential expression method used for prediction scoring. (classi, wilcox, t test). |
| Reference data for ScType | Immune system | Selected cell type reference for matching. |
| Top genes prediction GPTCelltype | 10 | Number of top genes used for GPTCelltype or other predictions. |
| Model for GPTCelltype | gpt-5 | OpenAI models available in GPTCelltype. gpt-5, gpt-5-mini, gpt-5-nano, gpt-4, gpt-4o, gpt-4-turbo, gpt-3.5-turbo, etc. |
| Use Own Labels | Cluster 0 to Cluster N | This option allows users to manually assign custom names to clusters. Users may enter identical names for two or more clusters if they wish to merge them into a single group |
| **Cluster-Based Plots** | | |
| No. of features to display | 3 | Number of genes to visualize per plot or select the list of gene names from the dropdown and type the specific genes which you are interested in eg: KLk2,KLK3,CTSG,MS4A3. |
| Select one or multiple cluster(s) for plotting | Default all clusters | User can adjust the cluster to plot. |
| Plot type | Spatial Plot | Types of visualizations for gene expression or differentially expressed genes. (Spatial plot, Dot Plot, Violin Plot, Ridge Plot, Feature Plot, Volcano Plot). |
| Dim plot labels | No | Whether to display labels in dimensionality reduction plots. |
| Group.by | condition | Grouping variable for DE or plotting, e.g., condition or sample ID. |
| Split.by | NULL | Whether to split plots by condition, sample, or not at all. |
| **Condition Based Plots** | | |
| Select the Condition1 | Group1 | User can select any one condition. |
| Select the Condition2 | Group2 | User can select any one condition. |
| min.pct | 0.25 | Minimum fraction of cells expressing the gene to be tested in marker analysis. Min: 0.01, Max: 1.0 |
| logfc.threshold | 0.25 | Log fold change threshold for identifying differentially expressed genes. Min: 0.01, Max: ∞ |
| Statistical test | wilcox | Test used for differential expression: e.g., wilcox, wilcox_limma, bimod, roc, t, LR, MAST. |
| Positive markers only | Yes | If Yes, return only genes upregulated in the target group. |
| group.by | condition | Metadata variable to group cells during marker analysis. (Condition and samples). |
| Plot type | Spatial Plot | Types of visualizations for gene expression or differentially expressed genes. (Spatial Plot, Dot Plot, Violin Plot, Ridge Plot, Feature Plot, Volcano Plot. |
| Number of features to display | 3 | Number of genes to visualize per plot or select the list of gene names from the dropdown and type the specific genes which you are interested in eg: KLk2,KLK3,CTSG,MS4A3 |
| **Subclustering** | | |
| Cluster Type Selection | Seurat clusters | Choose source for subclustering (Seurat, predicted, or gene-based selection. |
| Select cluster(s) | Select the cluster default 0 | Generated based on selected cluster type. |
| Genes to include (positive selection) | Eg: FCN1 or FCN1,PSAP | Enter comma-separated gene symbols for subsetting. |
| Genes to exclude (negative selection) | Eg: FCN1 or FCN1,PSAP | Enter comma-separated gene symbols to exclude cells. |
| **Correlation** | | |
| Input data | Output of single or multiple samples | Select the input from full dataset or subclustering. |
| Celltype method | Seurat clusters | Select celltype grouping for correlation. (Seurat clusters or predicted). |
| Correlation method | Spearman | Method to compute correlation between clusters (Pearson, Spearman, Kendall). |
| **Gene Ontology** | | |
| Input data | Output of single or multiple samples | Choose the source of genes for GO analysis. |
| Celltype method | Seurat clusters | Clustering source for gene selection. (Seurat clusters or predicted). |
| Organism | Human | Organism-specific annotation package. (Human, Mouse, Rat, Pig, Rhesus). |
| Ontology | BP | GO ontology cate.g.ories: biological process, etc. (BP, MF, CC, ALL). |
| pAdjustMethod | BH | Method for p-value adjustment. (holm, bonferroni, BH, BY, fdr, none). |
| pvalueCutoff | 0.05 | Significance threshold for raw p-value. Min: 0, Max: 1 |
| qvalueCutoff | 0.2 | Significance threshold for q-value. Min: 0, Max: 1 |
| Minimal gene size | 10 | Minimum number of genes in a category. Min: 1, Max: 500 |
| Maximal gene size | 500 | Maximum number of genes in a category. Min: 10, Max: 5000 |
| Plot type | Dotplot | Visualization options for enriched GO terms. (dotplot, barplot, cnetplot, upsetplot). |
| Top categories to plot | 10 | Number of categories to include in plots. Min: 1, Max: 50 |
| **Pathway Analysis** | | |
| Pathway analysis type | KEGG | Source of pathway database. (KEGG or Reactome). |
| Input data | Output of single or multiple samples | Choose the source of genes for pathway analysis. |
| Celltype method | Seurat clusters | Clustering source for gene selection. (Seurat clusters or predicted). |
| Organism | Human | Organism-specific annotation package. (Human, Mouse, Rat). |
| pAdjustMethod | BH | Adjustment for multiple testing. (holm, bonferroni, BH, BY, fdr, none). |
| pvalueCutoff | 0.05 | Significance threshold for raw p-value. Min: 0, Max: 1 |
| qvalueCutoff | 0.2 | Significance threshold for q-value. Min: 0, Max: 1 |
| Minimal gene size | 10 | Minimum number of genes in pathway. Min: 5, Max: 500 |
| Maximal gene size | 500 | Maximum number of genes in pathway. Min: 15, Max: 5000 |
| Plot type | Dotplot | Type of plot for pathway enrichment. (dotplot, barplot, cnetplot, upsetplot). |
| Top categories to plot (Pathway) | 10 | Number of enriched pathways shown. Min: 1, Max: 50 |
| **GSEA Analysis** | | |
| Input data | Output of single or multiple samples | Choose the source of genes for GSEA analysis. |
| Celltype method | Seurat clusters | Clustering source for gene selection. (Seurat clusters or predicted). |
| Organism | Homo sapiens | Species-specific gene set database. (Homo sapiens, Mus musculus). |
| MSigDB category | Curated gene sets (C2) | Gene set collection from MSigDB. (H, C1, C2, C3, C4, C5, C6, C7, C8). |
| ScoreType | std | Controls whether to score all, positive or negative enrichment. (std, pos, neg.). |
| Minimal gene size | 15 | Minimum genes per gene set. Min: 5, Max: 500 |
| Maximal gene size | 50 | Maximum genes per gene set. Min: 15, Max: 5000 |
| Permutations | 100 | Number of random permutations to compute significance. Min: 10, Max: 10000 |
| Plot type | GSEA plot | Style of plot for GSEA results. (GSEA plot, plotGseaTable, barplot). |
| Top significant results to plot | 10 | Number of enriched gene sets to plot. Min: 1, Max: 50 |
| **Cell-Cell Communication Analysis (Cell-chat)** | | |
| Input data | Output of single or multiple samples | Source of expression data for CellChat. |
| Celltype method | Seurat clusters | Cell grouping used in communication analysis. (Seurat clusters or predicted). |
| Organism | PPI.human | Organism-specific protein-protein interaction database. (PPI.human, PPI.mouse). |
| Min % cells expressed | 0 | Minimum percent of cells expressing ligand/receptor. Min: 0, Max: 100 |
| LogFC threshold | 0 | Minimum log fold change for expression filter. Min: 0, Max: 10 |
| P-value threshold | 0.05 | Significance cutoff for ligand-receptor pairs. Min: 0.0001, Max: 1 |
| Averaging method | triMean | Method for averaging gene expression per group. (triMean, truncatedMean, thresholdedMean, median). |
| Minimum cell count | 10 | Minimum number of cells in a group. Min: 5, Max: 1000 |
| Pattern k-value | 2 | Number of communication patterns to infer. Min: 2, Max: 20 |
| Show label | Yes | Display labels on communication plots. |
| Specific Signaling Pathways | The default 1st one is selected | Display the communication for the selected. |
| **Trajectory & Pseudotime Analysis (Monocle3)** | | |
| Input data | Output of single or multiple samples | Select the processed input source. |
| Celltype method | Seurat clusters | Grouping variable for pseudotime. |
| use_partition | No | Whether to use partitioned cell sets. |
| close_loop | Yes | Allow trajectory graph to close loops. |
| label_groups_by_cluster | No | Whether to show cluster labels. |
| label_branch_points | Yes | Show pseudotime branch points. |
| label_roots | Yes | Show root cells in trajectory. |
| label_leaves | No | Show leaf cells in trajectory. |
| Order cell in Pseudotime | Select one cluster as the root | Displays all clusters. |
| Gene functional change (neighbor_graph) | principal_graph | Graph type for trajectory inference. principal_graph, knn |
| Top genes to display in feature plot | 5 | Number or list of genes to plot along pseudotime. |
| **Co-expression & Network Analysis (hdWGCNA)** | | |
| Input data | Output of single or multiple samples | Select the processed input source. |
| Celltype method | Seurat clusters | Seurat cluster or predicted. |
| Select any one cluster | Default 0 | List all the clusters or names. |
| Input data | Output of single or multiple samples | Select the processed input source. |
| Reduction type | UMAP | Dimensionality reduction for module visualization. (UMAP or PCA). |
| Select soft-power Network type | signed | Type of WGCNA correlation network. (signed, unsigned, signed hybrid). |
| Module eigengenes and connectivity Scale model | linear | Statistical model for eigengene computation. (linear, poisson, negbinom). |
| Harmonized eigengenes | Yes | Whether to harmonize eigengenes across datasets. |
| Nearest neighbors (k) | 10 | K for building metacells. Min: 1, Max: 100 |
| Minimum cell group size | 10 | Minimum cells in a group to build a metacell. Min: 5, Max: 100 |
| Max shared cells | 15 | Max overlap between metacells. Min: 1, Max: 100 |
| Target metacells | 1000 | Max number of metacells to construct. Min: 50, Max: 5000 |
| Hub genes per module | 5 | Number of top hub genes labeled. Min: 1, Max: 50 |
| Show inter-module edges | No | Whether to draw edges across modules. |
| **Transcription Factor Regulatory Network Analysis (hdWGCNA)** | | |
| Organism | Human | Reference genome annotation. (Human or Mouse). |
| XGBoost max_depth | 1 | Tree depth for motif-based TF prediction. Min: 1, Max: 10 |
| eta | 0.1 | Learning rate in XGBoost. Min: 0.01, Max: 1 |
| alpha | 0.5 | Regularization parameter. Min: 0, Max: 1 |
| Regulatory score threshold | 0.01 | Minimum score for defining TF-gene edge. Min: 0, Max: 1 |
| Top TFs per gene | 10 | Top regulators retained per gene. Min: 1, Max: 50 |
| Positive regulon threshold | 0.05 | Minimum expression for positive regulons. Min: 0, Max: 1 |
| Negative regulon threshold | -0.05 | Threshold for defining negative regulons. Min: -1, Max: 0 |
| Color network edge by | Cor | TF network edge attribute. (Cor, Gain). |
| Extend TF network layers | Primary and secondary | Depth of TF-target extension. (Primary or Primary and secondary or Primary, secondary and tertiary). |
