# Supplementary material for: VST-DAVis: an R Shiny application and web-browser for spatial transcriptomics data analysis and visualization
Source: Bioinform Adv. 2026 Jan 9;6(1):vbag007. doi: 10.1093/bioadv/vbag007 (PMC12866912; doi:10.1093/bioadv/vbag007)
Supplement: vbag007_Supplementary_Data [file vbag007_supplementary_data.zip › Table_S3.docx]

**Table S3.** List of command-line tools and tutorials used to develop VST-DAVis. These tools perform tasks from initial QC to advanced analyses without the need for coding.

| Analysis | Tools | Hyperlink |
| --- | --- | --- |
| Single or Multiple Samples Analysis | Seurat | <https://satijalab.org/seurat/articles/visiumhd_analysis_vignette> |
| Cell Type Prediction | SingleR | <https://bioconductor.org/packages/release/bioc/vignettes/SingleR/inst/doc/SingleR.html> |
|  | GPTCelltype | <https://winnie09.github.io/Wenpin_Hou/pages/gptcelltype.html> |
|  | ScType | <https://github.com/IanevskiAleksandr/sc-type> |
| Correlation Network Analysis | genesorteR | <https://github.com/mahmoudibrahim/genesorteR/wiki/Visualize-single-cell-data-in-R-using-genesorteR-&-ggraph> |
| GO Term Analysis | clusterProfiler | <https://yulab-smu.top/biomedical-knowledge-mining-book/clusterprofiler-go.html> |
| Pathway Analysis | Pathway analysis | <https://yulab-smu.top/biomedical-knowledge-mining-book/clusterprofiler-kegg.html> |
|  | ReactomePA | <https://yulab-smu.top/biomedical-knowledge-mining-book/reactomepa.html> |
| GSEA Analysis | fgsea | <https://bioconductor.org/packages/release/bioc/vignettes/fgsea/inst/doc/fgsea-tutorial.html> |
| Cell-Cell Communication Analysis | Cellchat | <https://htmlpreview.github.io/?https://github.com/jinworks/CellChat/blob/master/tutorial/CellChat_analysis_of_spatial_transcriptomics_data.html> |
| Trajectory and Pseudotime Analysis | Monocle3 | <https://cole-trapnell-lab.github.io/monocle3/docs/getting_started/> |
| Co-Expression and TF Analysis | hdWGCNA | <https://smorabit.github.io/hdWGCNA/articles/basic_tutorial.html> |
|  | hdWGCNA TF | <https://smorabit.github.io/hdWGCNA/articles/tf_network.html> |
